# Identification and tissue expression profile of genes from three chemoreceptor families in an urban pest, *Periplaneta americana*

**DOI:** 10.1038/srep27495

**Published:** 2016-06-09

**Authors:** Yan Chen, Ming He, Zhao-Qun Li, Ya-Nan Zhang, Peng He

**Affiliations:** 1 Key Lab of Optimal Utilization of Natural Medicine Resources, Department of Pharmacology of Chinese Material Medica, Guizhou Medical University, Huaxi District, Guiyang, Guizhou 550025, People’s Republic of China; 2 State Key Laboratory Breeding Base of Green Pesticide and Agricultural Bioengineering, Key Laboratory of Green Pesticide and Agricultural Bioengineering, Ministry of Education, Guizhou University, Huaxi District, Guiyang 550025, People’s Republic of China; 3 Key Laboratory of Tea Biology and Resource Utilization, Ministry of Agriculture, Tea Research Institute, Chinese Academy of Agricultural Science, Hangzhou, 310008, People’s Republic of China; 4 College of Life Sciences, Huaibei Normal University, Huaibei, 235000, People’s Republic of China

## Abstract

*Periplaneta americana* is a notorious urban pest prevalent in human habitats; very little is known about its chemosensory mechanism. Employing the advanced next-generation sequencing technique, in the present study, we conducted transcriptome sequencing and analysis of the antennae of the adult males and females as well as their mouthparts using an Illumina platform. This resulted in the discovery of a huge number of the members of all major known chemosensory receptor families in *P. americana*, including 96 odorant receptors (ORs), 53 ionotropic receptors (IRs), and 33 gustatory receptors (GRs). Tissue expression profiles showed most of them mainly expressed in antennae and phylogenetic analysis demonstrated the expansion in the clade distinguishing them from other functionally well-known Lepidoptera species. A high percentage of chemosensory receptor genes (ORs in particular) showing female antenna bias in mRNA expression was observed. Our results provide a basis for further investigations on how *P. americana* coordinates its chemosensory receptor genes in chemical communication with environments, and for development of novel pest management approaches.

Insects communicate with their environment through chemosensation to help them accomplish a large number of essential physiological processes, such as mate-finding, host location, and alarming their conspecifics. Insects employ three major groups of chemosensory receptors, namely odorant receptors (ORs), variant ionotropic receptors (IRs), and gustatory receptors (GRs)^1^,^2^,^3^. Insect ORs and GRs possess seven transmembrane domains; compared to mammalian G-protein coupled receptors, orientation of these domains is reverse, with an intracellular N-terminus and an extracellular C-terminus^4^. Analysis of available insect genome sequences indicates that the OR family has undergone rapid evolution in a species-specific manner. A highly variable number of insect ORs have been identified in different insect species; for example, 62 ORs occur in *Drosophila melanogaster*^5^, 79 in *Anopheles gambiae*^6^, 170 in *Apis mellifera*^7^, 259 in *Tribolium castaneum*^8^, and 66 in *Danaus plexippus*^9^. In *D. melanogaster* antenna, only one OR gene is expressed in most dendritic membranes of the olfactory sensory neurons (OSNs)^10^. OR co-receptor (ORco), being one of the most conserved OR genes among various species, pre-dates other ORs that appear only in winged insects^11^. ORco interacts with other ligand-specific ORs to form an ORx-ORco complex, which functions as a ligand-gated cation channel^12^. Pheromone receptors (PRs), one of the sub-classes of ORs, are specifically activated by sex pheromone components and have been widely studied in Lepidoptera^13^,^14^. Not much is known about the recognition mechanism in other insect species with sex pheromones of different chemical structure.

GRs, the other members of the chemosensory superfamily, are likely to have the same ancestor as ORs and also have a seven transmembrane structure^15^. Insect GRs mainly respond to non-volatile ligands with the exception of CO_2_. Based on the known substances to which they respond, GRs were grouped with sugar receptors^16^, CO_2_ receptors^17^, and bitter or other receptor^18^. Although GRs do not have a common coreceptor such as ORco, they are also supposed to form heteromeric complexes, without fixed combinations^19^. IR, a new family of olfactory receptors, has the same ancestor as the mammalian ionotropic glutamate receptors (iGluRs)^20^. IRs are involved in insect odorant reception, as was first shown by the combined misexpression experiments and their subcellular localization in olfactory organs in *D. melanogaster*^20^. Further research showed that IRs differed from ORs in responding to carboxylic acids and amines^21^. IRs can be classified into two distinct subfamilies with different ancestors, namely, the conserved “antennal IRs” and the species-specific “divergent IRs.” The antennal IRs may represent the original olfactory receptor family of insects. *IR8a* and *IR25a*, belonging to antennal IRs, are two IR co-receptors^22^; they usually exhibit higher expression than other IRs^23^,^24^. Besides the olfactory function, IRs also play a key role in gustation. In *D. melanogaster*, *IR52c* and *IR52d* are co-expressed in the taste sensilla of the foreleg of males and regulate sexual behaviour, whereas *IR76b* is involved in salt tasting^25^.

Cockroaches are a big group of ancient insects of the order Blattodea. Of the approximately 4,600 cockroach species, about 30 are associated with human habitats. American cockroach, *Periplaneta americana*, one of the notorious urban pests is common and difficult to control. It can tolerate a wide range of environments, from Arctic cold to tropical heat. Periplanone-A and periplanone-B are two major sex-pheromone components emitted from the female *P. americana*, which trigger strong electroantennogram responses and behavioural response of conspecific male individuals^26^. To avoid toxic baits, cockroaches rapidly evolved their gustatory system responsible for glucose aversion^27^. However, little is known about the chemosensory mechanism of these essential chemicals (such as sex pheromone components, periplanone-A and periplanone-B, and glucose) in this species. Only few chemosensory receptor gene fragments have been identified in another cockroach species, *Blattella germanica*^28^. Therefore, in the present study, we sequenced and analyzed three transcriptomes of the adults of *P. americana* using next-generation sequencing, and identified 190 putative chemosensory receptor genes. These included 96 ORs, 61 iGluRs/IRs, and 33 GRs. To understand their potential functions, we conducted gene ontology (GO) annotation and scanned the tissue-specific expression of these sequences, as well. We also examined their phylogenetic relationships with some other insect species.

## Results

### Sequencing and assembly of transcriptome

Three transcriptomes from male antennae (mA), female antennae (fA), and mouthparts (Mo) were sequenced using the Illumina HiSeq™ 4000 platform (Illumina, Tianjin, China) and assembled with Trinity (version: r20140413p1)^29^. About 45.91 (mA), 43.45 (fA), and 43.17 (Mo) million reads were obtained for each transcriptome. After filtering, 44.45 (mA), 41.92 (fA), and 42.34 (Mo) million clean reads were generated, which comprised of 6.67 (mA), 6.29 (fA), and 6.35 (Mo) gigabases (Gb), with a longest unigene length of 32,380 nt and a median length of 327 nt after combined assembly of these three transcriptome. Finally, these reads were assembled into 304,023 transcripts and 248,192 unigenes, with N50 lengths of 1,155 and 795 nt, respectively (Table 1). In addition, the unigenes with a sequence length >500 nt accounted for 29.14% of the transcriptome assembly (Supplementary Fig. S1). The transcriptome raw reads have been deposited with the NCBI SRA database (accession number: SRR3089536, SRR3089537, and SRR3089538).

### Homology analysis and gene ontology annotation

**Figure 1:**
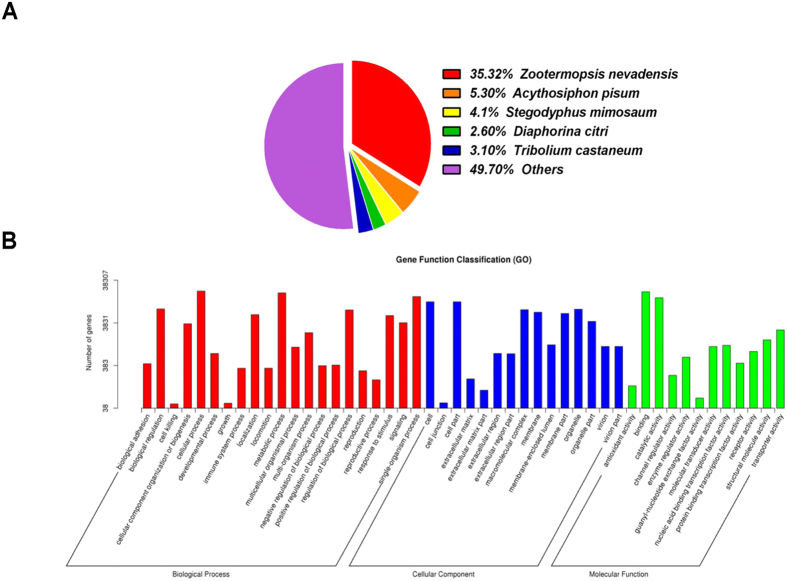
Annotation summaries for *P. americana* unigenes. (**A**) Species distribution of unigenes with the best hit annotation terms in the non-redundant (NR) database. (**B**) Gene ontology (GO) classifications of *P. americana* unigenes.

BLASTx homology searches of all the 248,192 unigenes showed that 40, 294 (16.23%) had homologous genes in the non-redundant (NR) protein database with a cut-off E-value of 10^−5^. The best match percentage (35.32%) was with *Zootermopsis nevadensis* sequences, followed by sequences from *Acyrthosiphon pisum* (5.30%), *Stegodyphus mimosaum* (4.10%), *T. castaneum* (3.10%), and *Diaphorina citri* (2.60%) (Fig. 1A).

Gene ontology (GO) annotations for all the unigenes were obtained using the Blast2GO pipeline, according to the BLASTx search against NR. The GO annotations were used to classify the transcripts into functional groups according to specific GO categories. Among the 248,192 unigenes, 38,307 (15.43%) could be assigned to various GO terms. In the molecular function category, the genes expressed in the three organs were mostly enriched for binding (e.g., nucleotide, ion, and odorant binding) and catalytic activity (e.g., hydrolase and oxidoreductase). In the biological process category, most common were the cellular and metabolic processes. In the cellular component terms, the most abundant were cell and cell part (Fig. 1B).

### Identification of *P. americana* OR/IR/GR genes

The unigenes related to candidate olfactory receptors (ORs/IRs/GRs) were identified based on the keyword searches of the BLASTx and Pfam family annotations. To avoid missing any *P. americana* OR/IR/GR genes, the predicted unigene protein sequences were also analyzed using PSI-BLASTp with OR/IR/GR sequences from *B. germanica*^30^ and *Z. nevadensis*^31^. In all, we identified 190 unigenes belonging to the chemosensory receptor family in the transcriptome of male antennae, female antennae, and mouthparts of *P. americana*. These included 96 ORs (including one ORco gene), 61 IRs/iGluRs (53 IRs and 8 iGluRs), and 33 GRs, all of which shared high identities with other insect OR (~34–88%), IR (~33–95%), and GR (~29–84%) genes after re-BLASTx identification. Information on the OR, IR, and GR genes including the unigene references, lengths, and the best BLASTx hits are listed in Supplementary Table S2.

### Tissue expression profile of *P. americana* OR/IR/GR genes

**Figure 2:**
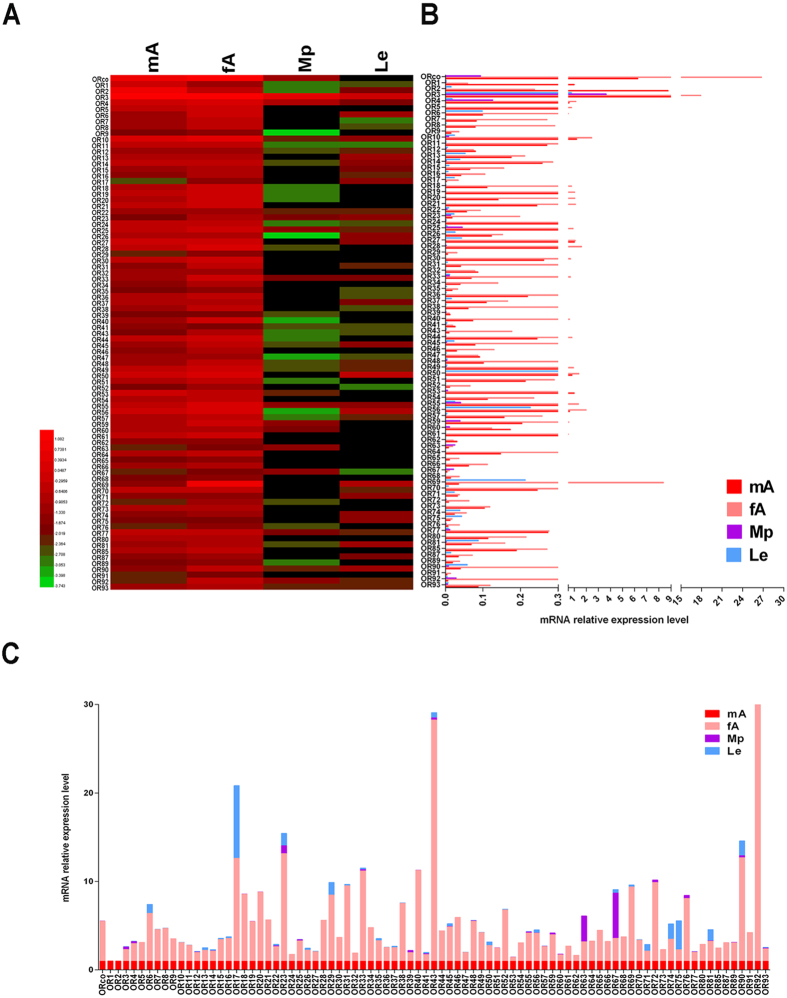
Tissue expression profile of *PameOR* genes based on relative mRNA quantity. (**A**) Heat map illustrating the Log_10_ transformation of mRNA expression levels of the *PameORs* in different tissues. (**B**) Relative mRNA expression level of all the *PameOR* genes; the level of *PameOR1* expression in the male antennae was set as 1. (**C**) Relative mRNA expression level of each *PameOR* gene in a represented stack; the level of *PameOR* in the male antennae was set as 1. mA, male antennae, fA, female Mp, maxillary palps, Le, Legs.

**Figure 3:**
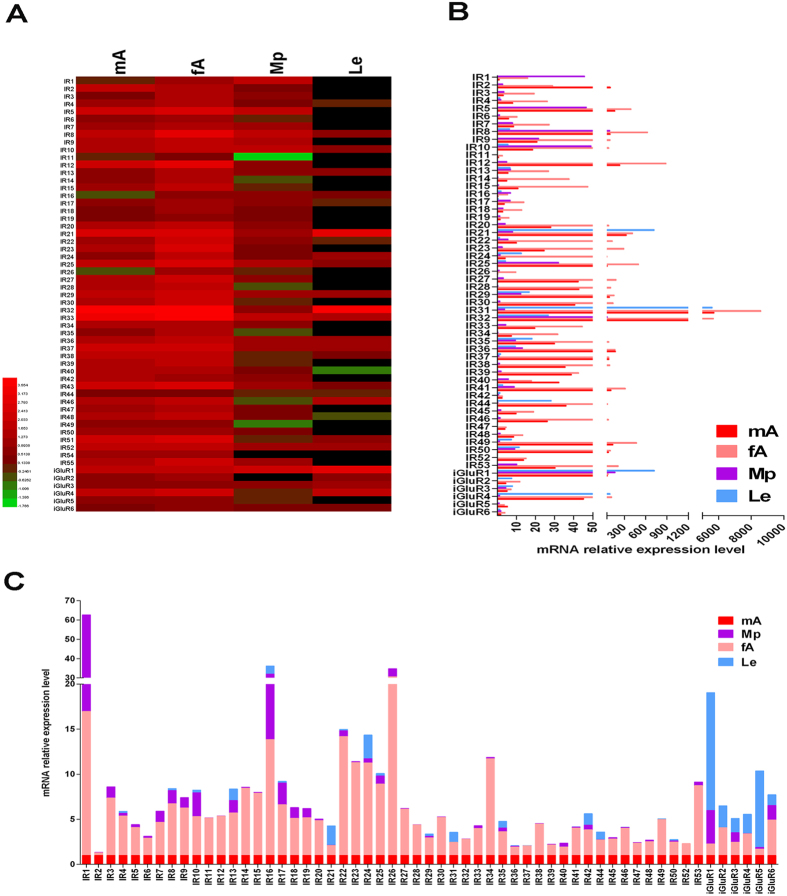
Tissue expression profile of *PameIR* genes based on relative mRNA quantity. (**A**) Heat map illustrating the Log_10_ transformation of mRNA expression levels of the *PameIRs* in different tissues. (**B**) Relative mRNA expression levels among all the *PameIR* genes; the expression level of *PameIR1* in the male antennae was set as 1. (**C**) Relative mRNA expression level of each *PameIR* gene in a represented stack; the relative expression of *PameIR* in the male antennae was set as 1. mA, male antennae, fA, female Mp, maxillary palps, Le, Legs.

**Figure 4:**
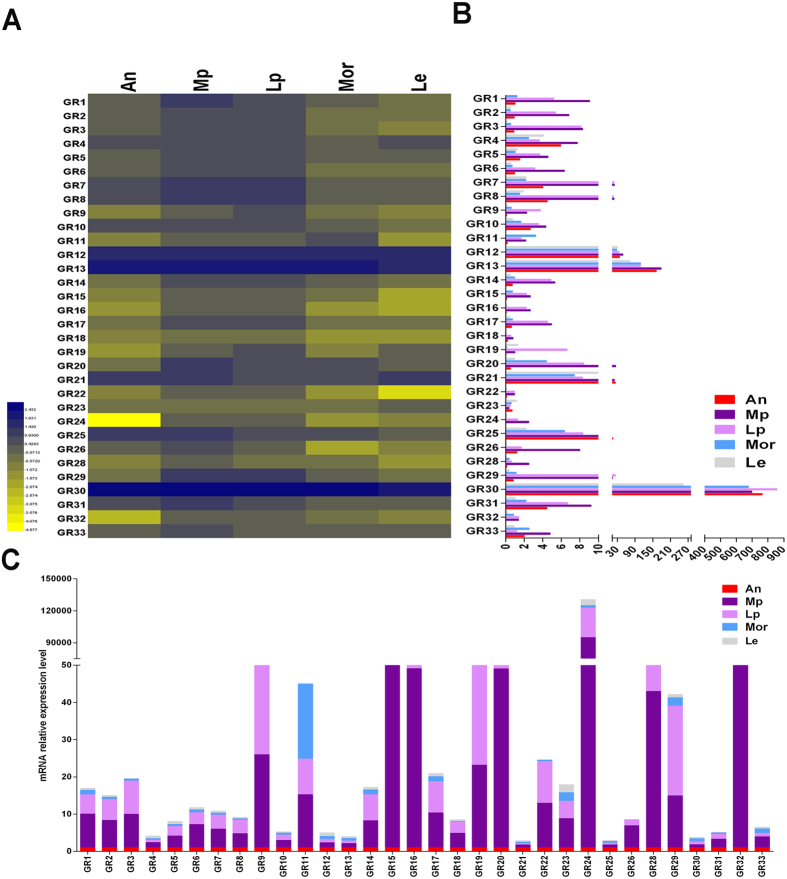
Tissue expression profile of *PameGR* genes based on relative mRNA quantity. (**A**) Heat map illustrating the Log_10_ transformation of mRNA expression levels of the *PameGRs* in different tissues. (**B**) Relative mRNA expression level of all the *PameGR* genes; the level of expression of *PameGR1* in the antennae was set as 1. (**C**) Relative mRNA expression level of each *PameGR* gene in a represented stack; the level of expression of *PameGR* in the antennae was set as 1. An, antennae, Mp, maxillary palps, Lp, labial palps Mor, mouthparts without Mp and Lp, Le, Legs.

Specific tissue expression pattern usually indicates specific gene functions. Thus, quantitative polymerase chain reaction (qPCR) was conducted to investigate the tissue expression profile of the candidate *P. americana* OR/IR/GR genes (Figs 2, 3, 4). For OR and IR genes, we selected four tissues, namely the male antennae, female antennae, maxillary palps (Mp), and legs (Le). The results indicated that these OR or IR-encoding genes were expressed exclusively in the antennae (especially in female antennae) of the olfactory organ. Based on statistical analysis, 79 of the 85 *P. americana* (*Pame*) *ORs* and 43 of the 46 *PameIRs* showed antenna-biased expression pattern (11 *PameORs* and 4 *PameIRs* were not amplified in all the tested tissues despite attempts using multiple primer pairs). Of these, *PameORco* had the highest expression among all the ORs. Only 4 *PameORs* and one *PameIRs* were dominantly expressed in the male antennae, 53 *PameORs* and 30 *PameIRs* were female antennae-biased, and 22 *PameORs* and 11 *PameIRs* were equal expressed in the male and female antennae (Figs 2 and 3). Due to their predominant expression in male antennae (the Male antennae/Female antennae mRNA expression level >10), two other highly expressing *PameORs*, *PameOR1*, and *PameOR2*, with expression levels in the male antennae being 16.8- and 36.9-fold higher than in the female antennae, respectively (Fig. 2A–C) were observed, suggesting the two ORs may participate in female sex pheromones recognition. Seven *PameORs*, *PameOR17, 23, 33, 40, 43, 90*, and *92*, three *PameIRs*, *PameIR23, 24*, *and 25* (Fig. 3A–C) demonstrated female-specific expression (Female antennae/Male antennae >10). We also found 2 *PameORs* (*PameOR63* and *67*) and 4 *PameIRs* (*PameIR1*, *10*, *16* and *17*) to be dominantly expressed in the major chemosensory organs not only in the antennae but also in the maxillary palps. However, 4 *PameORs* (*PameOR17*, *74*, *75*, and *81*), 4 PameIRs (*PameIR21, 31, 35* and *42*), and all 6 iGluRs (*PameiGluR1*~*PameiGluR6*) were not observed to have an obvious chemosensory organ-biased expression.

Five tissues were selected for investigation of *PameGR* genes: antennae mixed from both the sexes, maxillary palps, labial palps (Lp), mouthparts (Mor, without maxillary palps and labial palps), and legs. The results showed that 21 of the 32 *PameGRs* expressed majorly in the maxillary palps/labial palps (*PameGR27* was not detected in all the tested tissues; Fig. 4A–C); this included dominant expression of *PameGR20*, *26*, and *28* in the maxillary palps and labial palp-biased expression of *PameGR19*.

### Phylogenetic analysis

**Figure 5:**
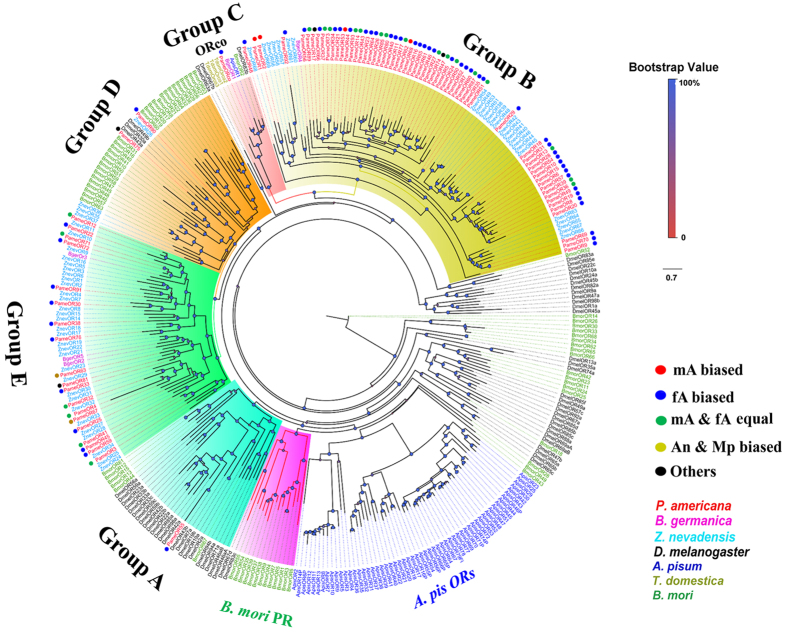
Phylogenetic tree of *P. americana* PameORs and other typical insect ORs. PameORs are highlighted in red. Species abbreviations: Apis, *A. pisum*; Pame, *P. americana*; Bger, *B. germanica*; Znev, *Zootermopsis nevadensis*; *Dmel, D. melanogaster*; Tdom, *Lygus lineolaris*; Bmor, *B. mori*.

To further uncover the functional role of *P. americana* OR/IR/GR genes, phylogenetic trees were constructed using sequences of typical OR/IR/GR genes from other insect species for which the whole genome or transcriptome data were available. Eighty-five PameORs were observed to be distributed in five major groups (Group A, B, C, D, and E) with other insect species ORs (Fig. 5). *Bombyx mori* PR and *A. pisum* OR groups were found to be independent groups without any orthologues of PameORs. Group C was the ORco group, which is the most conventional OR among various species; only one ORco gene, PameORco, was grouped in the same sub-group with another cockroach, B. germanica, BgerORco gene. PameOR55 was the only PameOR in Group A, which was grouped with 6 BmorORs and 23 DmelORs. PameOR75 and PameOR90 belonged to Group D with 29 BmorORs, 3 DmelORs, and 4 ZnevORs. Group B was a Blattaria-specific group with 62 PameORs, 26 ZnevORs, and 2 BgerORs. Group E was also a Blattaria-specific group that included 19 PameORs, 30 ZnevORs, and 3 BgerORs.

**Figure 6:**
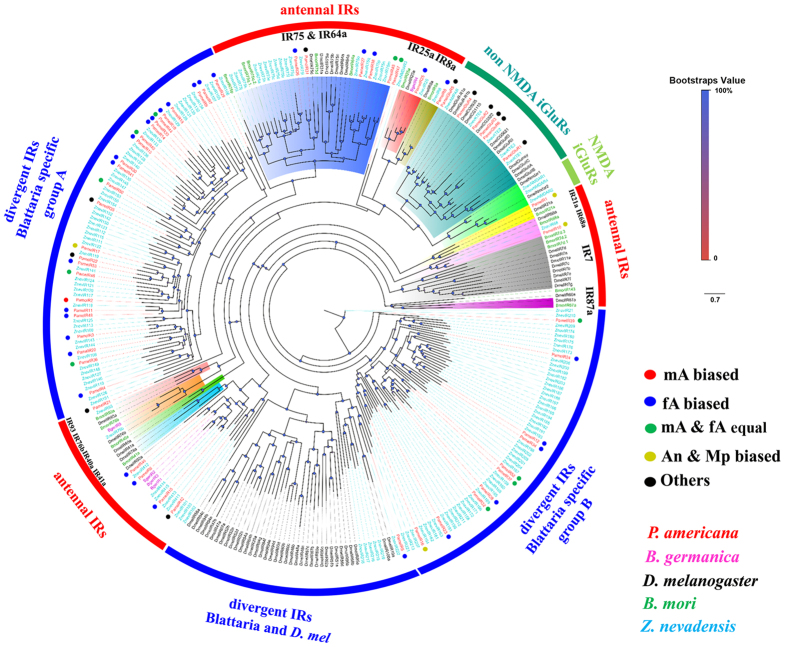
Phylogenetic tree of *P. americana* PameIRs and other typical insect iGluRs and IRs. *PameIRs* are highlighted in red. Bger, *B. germanica*; Znev, *Zootermopsis nevadensis*; *Dmel, D. melanogaster*; Bmor, *B. mori*.

Four major iGluR groups were the antennal IR, divergent IR, NMDA iGluR, and non-NMDA iGluR groups. IR8a and IR25a were co-receptors present in the IR group. Two PameIRs, PameIR8 and PameIR25, were observed to be distributed in these two co-receptors groups, respectively. Six PameIRs, PameIR26, 27, 31, 38, 51, and 53, were distributed in the IR75 and IR64a groups. PameIR1 and PameIR10 are the orthologous genes of antennal IR21a and IR68a groups, respectively. PameIR6, 15, 44, and 48 were four genes that were grouped with the antennal IR41a. However, no orthologous genes of IR87a, IR7, IR41a, IR93, and IR76b were found in *P. americana*. Two major divergent IR groups of *Blattaria* were found in the IR tree, namely the A and B group. Group A contained 26 PameIRs and 49 ZnevIRs. Group B contained 11 PameIRs and 48 ZnevIRs (Fig. 6).

**Figure 7:**
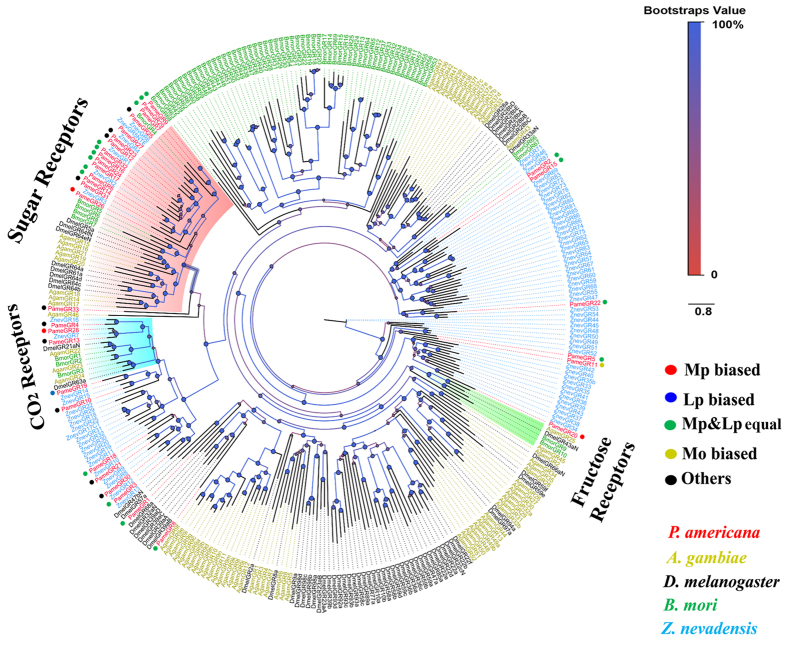
Phylogenetic tree of *P. americana* PameGRs and other typical insect GRs. *PameGRs* are highlighted in red. Bger, *B. germanica*; Znev, *Zootermopsis nevadensis*; *Dmel, D. melanogaster*; Bmor, *B. mori*, Agam, *A. gambiae*.

In the GR phylogenetic tree, 16 PameGRs and 3 PameGRs were distributed in the sugar and CO_2_ receptors, respectively (Fig. 7). No orthologues of fructose receptors were noticed. The other 12 PameGRs were grouped in another clade “bitter/other receptors” without functionally identified orthologues.

## Discussion

In the present study, we determined the repertoire of chemosensory receptor families (ORs, IRs, and GRs) in a notorious urban pest, *P. americana*. These receptor genes have potential significance as targets for developing new pest control strategies, as well as for elucidating the molecular mechanisms that underlie insect-host interactions. Transcriptomes (19.31 Gb in total) of three chemosensory organs, male antennae, female antennae, and mouthparts, were sequenced; this was higher than the transcriptomes processed in most of the other studies^32^,^33^,^34^. After extensive sequencing and assembly using the Trinity software, we identified 96 ORs, 61 IRs/iGluRs (53 IRs and 8 iGluRs), and 33 GRs in this species. The number of ORs was much higher than in the two other *Blattaria* species, *Z. nevadensis* (60 ORs, genome data)^31^ and *B. germanica* (5 ORs, antennal transcriptome data)^30^. It was also higher than the 62 ORs found in *D. melanogaster*^15^ and the 79 ORs in *A. gambiae*^6^,^35^, but was much lower than the number of ORs reported in *T. castaneum* (259 ORs)^8^, *A. mellifera* (170 ORs)^7^, and *L. moratoria* (142 ORs)^36^. The number of IRs was lower than the numbers found in *Z. nevadensis* (136 IRs)^31^ and higher than those found in *B. germanica* (5 IRs, antennal transcriptome data)^30^, but it was much higher than the 14 IRs found in *A. gossypii*^24^ and *Sogatella furcifera*^23^, 57 in *D. melanogaster*^20^,^22^,^37^, and 22 in *A. gambiae*^38^. The number of GRs was much lower than that in *Z. nevadensis* (75 GRs)^31^, *D. melanogaster* (68 GRs)^15^, *Anopheles gambiae* (72 GRs)^6^, and *B. mori* (65 GRs)^39^, but was higher than in *A. mellifera* (13 GRs)^7^. Unlike other ancient insects, which either lack *ORco* (*Lepismachilis y-signata*) or possess three *ORco* genes (*Thermobia domestica*)^11^, only one *ORco* gene was found in *P. americana*, which has implies a fully developed ORx/ORco-based olfactory system. This result is same as that observed in another cockroach, *B. germanica*, where also only one *ORco* gene, *BgerOR1*, an orthologue of *PameORco*, was present in the antennal transcriptome^30^. *IR8a* and *IR25a* are also coreceptors in the IR system^20^. *PameIR8* and *PameIR25* were found clustered in the *IR8a* and *IR25a* group with members from other insect species, which are candidate IR coreceptors in *P. americana* and the orthologous genes, *ZnevIR8*, *ZnevIR25*, and *BgerIR4* for the IR coreceptors from two other *Blattaria* species. These findings suggest that the adaptation of distinct species to their hosts has led to the diversification of ORs, IRs, and GRs during their evolution.

In some lepidopteran species, the expressions of ORs in male antennae are mostly higher than or equal to that in female antennae; for example, in *Spodoptera litura*, 17 of the 25 ORs are male antenna-biased and only 2 of 25 ORs show female-biased expression^40^. In contrast, in *B. mori*, only 10/47 of the ORs (female/male >2) show a female-biased expression^41^. In *P. americana*, a lot of *PameORs* (53 out of 85) and *PameIRs* (30 out of 57) have a female antennae-biased expression; this was in contrast to the findings in *M. sexta* (7/70, RNAseq data, male/female >10)^42^ and *B. mori* (2/68, RT-PCR data, female specific)^43^. In another study on *B. germanica*, the authors selected two ORs, *BgerOR1* and *BgerOR2*, and one IR, *BgerIR5*, to investigate their tissue expression pattern by qPCR. All these three genes displayed female antenna-biased expression^30^. However, this female antenna-biased expression pattern is expected, as in some hymenopteran species, such as the two parasitoid wasps, *Chouioia cunea*^44^ and *Microplitis mediator*^45^, ORs and IRs have been reported to play a crucial role in female individuals for finding suitable host to lay eggs. However, cockroaches, which are the early ancestors, lack the internal ovipositors. Thus, we hypothesize that the female adults would find more food sources, using food volatiles as cues for the olfactory receptors, to meet the energy requirements of pregnant or egg-carrying females. Consistent with the roles of OR/IR in olfaction, most *Locusta migratoria* OR/IR genes displayed olfactory tissue specific expression. However, our results showed that Pame OR/IR genes not only exhibited antenna-biased expression patterns, but also antennae/maxillary palps-biased and other expression patterns. Two *PameORs* (*PameOR63* and *PameOR67*) and four *PameIRs* (*PameIR1*, *10*, *16*, and *17*) were expressed both in the antennae and maxillary palps, indicating that they might play key roles not only in olfaction but also in gustation, as previously reported in mosquito^46^ and fly^47^. Moreover, 4 *PameORs* (*17, 74, 75*, and *81*) and 4 *PameIRs* (*21, 31, 35* and *42*, and six iGluRs (*iGluR1–6*) exhibited non-chemosensory organ-biased expression. All of them expressed in the legs to a certain extent, which was consistent with some previous studies^36^,^47^,^48^. However, we could not ignore their olfactory function; as reported earlier, IR expression in the legs affected the mating behaviour of fly^47^.

*PameGRs* expressed mainly in the gustatory organs Mp and Lp, which was consistent with the findings in *D. melanogaster*^49^. Insect GRs have been classed in four groups: CO_2_-, fructose-, non-fructose sugar- receptors, and bitter/other receptors^50^. Based on the analysis of phylogenetic tree, we could assign a functional group to most of the *PameGRs*. The CO_2_ receptors are relatively conserved among the insect species^51^. Three putative CO_2_ receptors, *PameGR4*, *13*, and *28* were identified in *P. americana*. However, only *PameGR28* displayed maxillary palps-biased expression, indicating that it had a greater possibility to function as a CO_2_ receptor than the other two putative receptors. They exhibited apparent orthologous relationships with the *Z. nevadensis* GR genes, *PameGR4*/*ZnevGR16* and *PameGR13*/*PameGR28*/*ZnevGR7*. The presence of three CO_2_ receptors was consistent with the number of these receptors in most other insect species, except *D. melanogaster*, which only has two receptors, *DmelGR21a* and *DmelGR63a*^51^. The sugar receptors (SRs) were first reported in *D. melanogaster*. Eight genes, *DmelGR5a*, *64a-f*, and *61*, which are active for a number of non-fructose sugars *in vivo* by coding for a single GR or for heterodimers^19^. Fourteen putative SRs were identified in *P. americana*, which were present in same group in the phylogenetic tree as the *DmelSRs*. The number of SRs is variable in different insect species due to the distinct host and food they have; two have been reported in *A. mellifera*^7^, five in *M. sexta*^42^, *B. mori*^39^, and *Z. nevadensis*^31^, eight in *D. melanogaster*^19^ and *A. gambiae*^6^*, 10 in D. plexippus*^42^, and 16 in *T. castaneum*^52^. Eleven of the 14 SRs in *P. americana* showed Mp/Lp-biased expression pattern, including equal expression of 10 SRs in Mp and Lp. One SR was predominantly expressed in Mp, indicating the Mp/Lp are both important organs to sense sugars. Consistent with this observation, the sugar-binding sites are present in contact with chemosensory hairs on the maxillary palps of *P. americana*^53^. Four pairs of orthologues were observed, namely *PameGR7*/*PameGR9*/*PameGR29*/*BmorGR4*, *PameGR25*/*ZnevGR6*/*ZnevGR5*, *PameGR12*/*PameGR23*/*ZnevGR1*, and *PameGR6*/*ZnevGR2*. However, the functional information about these orthologous genes is not available. The other 15 *PameGRs* remain clustered with the predicted bitter/other receptors. There are several lineage-specific expansions of *Blattaria* species; the *P. americana* and *Z. nevadensis* bitter/other receptors are common with bitter receptors of other insect species^39^,^42^,^54^. Eleven orthologous gene pairs (*ZnevGR16*/*PameGR4*, *ZnevGR7*/*PameGR13*/*PameGR28*, *ZnevGR14*/*PameGR19*, *ZnevGR15*/*PameGR10*, *ZnevGR18*/*PameGR11*, *ZnevGR10*/*PameGR21*, *ZnevGR8*/*PameGR30*, *ZnevGR12*/*PameGR3*, *ZnevGR34*/*PameGR20*, *ZnevGR47*/*PameGR22*, and *ZnevGR69*/*PameGR2*/*PameGR15*) were observed in these two species indicating that the bitter/other receptors are conserved. *BmorGR9* in *B. mori*^55^ and *DmelGR43a* in *D. melanogaster* were defined as fructose receptors^16^. It has recently been reported that *DmelGR43a* can respond to a range of sugars^19^. However, we did not find any fructose receptor orthologue in *P. americana*, or in the genome of other known species, *Z. nevadensis* and *A. pisum*. We hypothesize that some other GRs, not present in this group, might play a role in detecting fructose.

Adults of female *P. americana* release two major active sex pheromone components, periplanone-A and periplanone-B, to solicit the male adults^26^. Thus, like in the lepidopterans, male antenna predominate expression is the standard to find PR^13^,^56^. *PameOR1* and *PameOR2*, the higher expressing *PameORs* in the same clade, are proposed to be the candidate PRs, with their expression levels in the male antennae being much higher than in the female antennae and feebly detected in other tissues. Certainly, the putative PR function needs further elucidation by *in vivo* and *in vitro* studies. Besides ORs, IRs, such as *IR52c* and *IR52d* of *D. melanogaster*, are also involved in the mating behaviour^47^. In *P. americana*, we observed only one IR, *PameIR2*, to possess male-antennae dominant expression. This IR belonged to the divergent IR of the *Blattaria*-specific group A with no orthologous genes, and far separated from *DmelIR52c* and *Dmel52d*. However, whether the two major sex pheromone components activate the two respective PRs or *PameIR2* needs to be investigated further.

In conclusion, based on the analyses of transcriptomic data, we identified a large number totally 190 new chemosensory receptors in the urban pest, *P. americana*; these included 96 ORs, 61 iGluRs/IRs, and 33 GRs, which is much number than many other insect species. Our results provide a valuable resource for investigating and elucidating the mechanisms of olfaction and gustation in *P. americana*. As a crucial first step towards understanding of their functions, we also conducted a comprehensive examination of the tissue expression patterns and phylogenetic tree of these olfactory receptor genes, which demonstrated that most of these OR, IR, and GR genes were expressed in the chemosensory organs and most OR and IR genes are female antenna-biased expression indicating their key role in female physiological behaviours. Our findings provide the foundation for future research into the olfactory and gustatory system of *P. americana*, such as the calling behaviour. It could also help in identifying a large number of potential target genes for controlling this notorious pest.

## Methods

### Insect samples

*P. americana* were purchased from an insect rearing factory in Anhui province, China. We collected 200 antennae and mouthparts each, from the adults of both the sexes (male/female = 1:1) for transcriptome sequencing. We dissected various tissues (male/female = 1:1, approximately 100 antennae of both the genders, 80 maxillary palps, 80 labial palps, and 80 mouthparts without maxillary palps and labial palps, and 100 legs for each replicate experiment) collected from the adults under a microscope in three replicates for each tissue type. The tissue samples were stored in liquid nitrogen at −80 °C until further use.

### cDNA library construction and Illumina sequencing

Total RNA was extracted using TRIzol reagent (Invitrogen Carlsbad, CA, USA) according to the manufacturer’s protocol. RNA degradation and contamination was monitored on 1% agarose gels. RNA purity was checked using the NanoPhotometer spectrophotometer (IMPLEN, CA, USA). RNA concentration was measured using Qubit RNA Assay Kit in Qubit 2.0 Fluorometer (Life Technologies, CA, USA). RNA integrity was assessed using the RNA Nano 6000 Assay Kit of the Agilent Bioanalyzer 2100 system (Agilent Technologies, CA, USA). The cDNA library construction and Illumina sequencing of the samples were performed by Novogene Bioinformatics Technology Co. Ltd., Beijing, China. A total amount of 3 μg RNA was used as input material for the RNA sample preparations. Sequencing libraries were generated using NEBNext Ultra RNA Library Prep Kit for Illumina (NEB, USA) following manufacturer’s recommendations and index codes were added to attribute sequences to each sample. Briefly, mRNA was purified from total RNA using poly-T oligo-attached magnetic beads. Fragmentation was carried out using divalent cations under elevated temperature in NEBNext First Strand Synthesis Reaction Buffer (5×). First strand cDNA was synthesized using random hexamer primer and M-MuLV Reverse Transcriptase (RNase H-). Second strand cDNA synthesis was subsequently performed using DNA Polymerase I and RNase H. Remaining overhangs were converted into blunt ends via exonuclease/polymerase activities. After adenylation of 3′ ends of DNA fragments, NEBNext Adaptor with hairpin loop structure were ligated to prepare for hybridization. In order to select cDNA fragments of preferentially 150–200 bp length, the library fragments were purified with AMPure XP system (Beckman Coulter, Beverly, USA). Thereafter, 3 μL USER Enzyme (NEB, USA) was added to size-selected, adaptor-ligated cDNA and incubated at 37 °C for 15 min followed by 5 min at 95 °C before PCR. PCR was performed with Phusion High-Fidelity DNA polymerase, Universal PCR primers, and Index (X) Primer. Finally, the PCR products were purified (AMPure XP system) and the library quality was assessed on an Agilent Bioanalyzer 2100 system.

### *De novo* assembly of short reads and gene annotation

Raw data (raw reads) in the fastq format were first processed through in-house perl scripts. In this step, clean data (clean reads) were obtained by removing the reads containing adapter, reads containing ploy-N, and low quality reads from the raw data. Simultaneously, Q20, Q30, GC-content, and sequence duplication level of the clean data were calculated. All the downstream analyses were based on clean data with high quality. *De novo* transcriptome assembly was performed using the short reads assembly program Trinity (version: r20140413p1) with min_kmer_cov set to 2 by default and all other parameters were set to default values. The overlap settings used for the assembly were 30 bp and 80% similarity, and all the other parameters were set to their default values.

Unigenes >150 bp were aligned by BLASTx with protein databases, including Nr, Swiss-Prot, KEGG, and COG (e-value <10^−5^), to identify proteins with high sequence identity and to assign putative functional annotations. Next, we used the Blast2GO program (version: b2g4pipe_v2.5, e-value = 1.0E-6) (https://www.blast2go.com/) to obtain GO annotations of the unigenes and we obtained the GO functional classifications using WEGO software (http://wego.genomics.org.cn/cgi-bin/wego/index.pl).

### Phylogenetic analysis

Amino acid sequences of the selected ORs, iGluRs/IRs, and GRs were aligned with the MAFFT (E-INS-I parameter)^57^. Thereafter, PhyML 3.1 with LG substitution model was used to construct a maximum likelihood phylogenetic tree using Bayesian analysis. The OR dataset comprised ORs in the available databases from: *A. pisum*^58^,^59^, *B. germanica*^30^, *D. melanogaster*^15^, *B. mori*^41^, *Z. nevadensis*^31^, and *T. domestica*^11^. The IR dataset comprised IRs from *B. germanica*^30^, *B. mori*^37^, as well as IRs and iGluRs from the model insect *D. melanogaster*^37^. The GR dataset comprised GRs from *D. melanogaster*^15^, *A. gambiae*^6^, *Z. nevadensis*^31^, and *A. mellifera*^7^. Finally, the trees were viewed and group edited with FigTree v1.4.2 (http://tree.bio.ed.ac.uk/software/figtree/).

### RNA extraction and cDNA synthesis

Total RNA was extracted using EasyPure RNA Kit (TransGen Biotech, Beijing, China) following the manufacturer’s instructions, in which DNase digestion was included to avoid the genomic DNA contamination. RNA quality was checked with a spectrophotometer (NanoDrop 2000, Thermo Fisher Scientific, USA). The single-stranded cDNA templates were synthesized from 1 μg of total RNA from various tissue samples using the PrimeScriptRT Master Mix (TaKaRa, Dalian, China) at 42 °C for 1 hr. The reaction was terminated by heating at 70 °C for 15 min.

### Quantitative real time PCR and data analysis

qPCRs were performed for each sample using an iCycle iQ (Bio-Rad, CA, USA) according to the minimum information for publication of quantitative Real-Time PCR Experiments^60^. Gene-specific primers were designed by Beacon Designer 7.6 (PREMIER Biosoft International, CA, USA) and are listed in Supplementary File 1. The mRNA levels were measured in triplicate (technical replicates) by qPCR using TransStart Tip Green qPCR SuperMix, as described by the manufacturer (TransGen Biotech, Beijing, China). The mRNA levels were quantified using ADP-ribosylation factor (the cDNA sequence was identified from this study), actin (Genbank accession number: AY11670), and 60S ribosomal protein L17 (the cDNA sequence was identified from this study) as the reference genes. Means and standard errors were obtained based on at least two biological replicates. The relative expression level of the mRNAs for OR, IR, and GR genes were calculated according to the 2^−ΔΔCq^ method. The relative fold-changes in the different tissues were calculated and normalized based on the transcript levels in the male antennae.

### Statistical analysis

Data (mean ± SE) from various samples were subjected to one-way nested analysis of variance (ANOVA) followed by a least significant difference test (LSD) for mean comparison using SPSS Statistics 17.0 (IBM, Chicago, IL, USA).

## Additional Information

**How to cite this article**: Chen, Y. *et al*. Identification and tissue expression profile of genes from three chemoreceptor families in an urban pest, *Periplaneta americana*. *Sci. Rep*. **6**, 27495; doi: 10.1038/srep27495 (2016).

## Supplementary Material

Supplementary Information

Supplementary File 1

## Figures and Tables

**Table 1:** Summary of *P. americana* transcriptome assembly.

Tissues	♂Antennae	♀Antennae	Mouthparts
Total size	6.67 Gb	6.29 Gb	6.35 Gb
GC content	36.80%	36.82%	41.15%
Number of transcripts	304,023		
Total unigene count	248,192		
Genes with homologues in NR	40,294		
Total transcript nucleotides	210,868,904		
Total unigene nucleotides	143,462,349		
N50 transcript length	1155 nt		
N50 unigene length	795 nt		
Longest unigene length	32,380 nt		
Median unigene length	327 nt		
