# Supplementary material for: Identification and tissue expression profile of genes from three chemoreceptor families in an urban pest, Periplaneta americana
Source: Sci Rep. 2016 Jun 9;6:27495. doi: 10.1038/srep27495 (PMC4899716; doi:10.1038/srep27495)
Supplement: Supplementary Information [file srep27495-s1.pdf]

# **Identification and tissue expression profile of genes from three chemoreceptor families in an urban pest, *Periplaneta Americana***

Yan Chen[1#], Ming He[4#], Zhao-Qun Li[3], Ya-Nan Zhang[2*], Peng He[4*]

[1] Key Lab of Optimal Utilization of Natural Medicine Resources, Department of Pharmacology of Chinese Material Medica, Guizhou Medical University, Guiyang, Guizhou 550025, People's Republic of China

[2] College of Life Sciences, Huaibei Normal University, Huaibei, 235000, P. R. China

[3.] Key Laboratory of Tea Biology and Resource Utilization, Ministry of Agriculture, Tea Research Institute, Chinese Academy of Agricultural Science, Hangzhou 310008, China

[4] State Key Laboratory Breeding Base of Green Pesticide and Agricultural Bioengineering, Key Laboratory of Green Pesticide and Agricultural Bioengineering, Ministry of Education, Guizhou University, Huaxi District, Guiyang 550025, P. R. China.

## Supplementary information

**Supplementary information** accompanies this paper at http://www.nature.com/srep

**Table S1. qPCR primers for tissue expression profiling.**

**Figure S1. Distribution of transcript and unigene lengths in the *P. americana* transcriptome assembly.**

**Table S2 Dataset 1. BLASTX results and sequence information of putative OR, IR, and GR in *P. americana*.**

**Table S1. qPCR primers for tissue expression profiling.**

| Gene Name | 5'→3' | Gene Name | 5'→3' |
|---|---|---|---|
| PameORco-F | TCACCTGAGAGCATAACTAAT | PameORco-R | GAGAATCCAAGCAGAATCG |
| PameOR1-F | CAGCGTGCTATAATGTTCAT | PameOR1-R | GACTATTGCCTTCGTGTTC |
| PameOR2-F | GCTGTATTGATGATGCTGAT | PameOR2-R | GGAGTGTTTCTTGCTATTCTT |
| PameOR3-F | GACAGACAAGCAAGTTACC | PameOR3-R | GCAGTTGATTCTAAGGAGTG |
| PameOR4-F | TTATAGTGACACGCTGGAA | PameOR4-R | GCACGCTACTCTAGTTCT |
| PameOR5-F | AGGAGGAGATATGATGTTGAA | PameOR5-R | TGCGTTGGAGTTCTATGT |
| PameOR6-F | TTACGAGTTGCTTGTTACATT | PameOR6-R | ACATTGGACCTGAGATGAA |
| PameOR7-F | CAGAGTTGTCCTTGTATATGTAG | PameOR7-R | TCAGCGTGAATCCTTGAT |
| PameOR8-F | CCTATGTAGTCAGTCAGATGT | PameOR8-R | CTATTCAATGGCGGCTATG |
| PameOR9-F | CGTTATCTTATCAGCGTTATCT | PameOR9-R | ATCTTCCGTGTTCATATCCT |
| PameOR10-F | ACGCACTTCCAGAGATAG | PameOR10-R | TCCGACAGTATTACACATAAAC |
| PameOR11-F | ATTCCAGCCATCATTAGACT | PameOR11-R | TAGTGCGACATCATCAAGA |
| PameOR12-F | ATCACCAGGCGATTATCA | PameOR12-R | CTGAAGAAGTAGAAGAGAAGAAG |
| PameOR13-F | GCACATCAAGTCCTCAATAC | PameOR13-R | CTTCATCCCACACCAGTA |
| PameOR14-F | CTTGCCTTATCAGCGATG | PameOR14-R | GTAACATTAGAGACTGGAACAA |
| PameOR15-F | TAATGACTGCTGGTAATTGTG | PameOR15-R | ATCTAATCTTCCGACTAAATGAC |
| PameOR16-F | ATCTTATCAGCGTTCCATTG | PameOR16-R | GTTCAGTCTATATTCTCAGTTCTT |
| PameOR17-F | CTATCAGCGATGTATATTGTTCA | PameOR17-R | ATTCATTGCGTCAGTTCTC |
| PameOR18-F | ACCTGACAGAATGTATTTGAC | PameOR18-R | AGTTCCTATCACAAGGCTAA |
| PameOR19-F | TCATATTGACGGCTGGAA | PameOR19-R | AGAAGAAACATAAAGAGAGACATT |
| PameOR20-F | AGTAACACGCTGAATGGTA | PameOR20-R | CAAACCCAACACATTACAGA |
| PameOR21-F | GGTATTTAGACGCTTTGGAAT | PameOR21-R | ACAGTGAGACTTGAAGAACA |
| PameOR22-F | GCTGAGTCCTGGTTATGA | PameOR22-R | TTCTGATTGGTCCCTTCTT |
| PameOR23-F | AAGGTTGTTCCAGTGTCT | PameOR23-R | TACTTCGTCATTGATTGATACTAC |
| PameOR24-F | CCGATCTCCAATTCTACTCTA | PameOR24-R | TTCACAATTCGTTGCTTCA |
| PameOR25-F | TTGCTGTGCGAGTTCTAT | PameOR25-R | AGGCTGGGTCTTTGTATC |
| PameOR26-F | TTGGAACTCTATTATCTGATGTG | PameOR26-R | TGTAGAATTGGAGACTGGAA |

| | | | |
|---|---|---|---|
| PameOR27-F | GACACCCACCTTCTCAAA | PameOR27-R | CACTCCTCAATCTTATGTAACAA |
| PameOR28-F | CACCATTCTACGCTACTCT | PameOR28-R | ATTGTCCATCACCGTCTT |
| PameOR29-F | TGGATAGGCTGTTCTACTTC | PameOR29-R | CACATATTCTTCTCTTCATTCTTG |
| PameOR30-F | TGGAAGGACTCGCTAATC | PameOR30-R | GCTGTATAACTGTGAAGTAAAGA |
| PameOR31-F | AAACAACGAGAAATAGAAAGGT | PameOR31-R | CTCTGAGAATTATCTTACAGTCTT |
| PameOR32-F | TATACTGCGTGATGGAGAC | PameOR32-R | AATGACAATGTGCCGAAC |
| PameOR33-F | CCTTTCTTCCATCTTTGTTGTA | PameOR33-R | AAGCAATTAACGAGGAGTAAC |
| PameOR34-F | TAGTAACAGATAAGAACGAGAGT | PameOR34-R | GGTAGAATTAGATAACGGAACAA |
| PameOR35-F | TATCTTTCCCTGGGTTCTTT | PameOR35-R | TCGCTTCTGATTCTGTGT |
| PameOR36-F | ACTTCATCAACAGACAATCAG | PameOR36-R | GCGAGAGCAATATTATTAGGAA |
| PameOR37-F | TCAGCGGTGTATCTTGTT | PameOR37-R | GTTCTTCATTCTTGTCCTTCA |
| PameOR38-F | GTTGTCGGTTGGAGTAATG | PameOR38-R | TGTAGCAGAATCGTGAAGTA |
| PameOR39-F | CAGGTTATCATGCTCACAAT | PameOR39-R | TGAATCGGTTATGGACAGA |
| PameOR40-F | CGAGTCAGTAAGTGAAGGA | PameOR40-R | AGTAGAATTGGACACAGGAA |
| PameOR41-F | GACCTGTGTTGCTGTTTG | PameOR41-R | GACTGTGATATTGCTCTGAATT |
| PameOR42-F | CTGGTATTGTAGTTAGTGTTCAT | PameOR42-R | ATGGTGACTTGATGGATACA |
| PameOR43-F | GCATCCTCTTCATCATAGC | PameOR43-R | CTCCGACTGTGGTAATCA |
| PameOR44-F | CGTCATAAGGCTCAATGTTAA | PameOR44-R | CGTTTCAGTGCTCTCAAAT |
| PameOR45-F | TTGCCTTCATTGTGATTGG | PameOR45-R | AGAACATTCCTTGCTCTACTA |
| PameOR46-F | TTATCTCACTGCTGGGAAG | PameOR46-R | TTACATTCAATCATCGCTCTTAT |
| PameOR47-F | TTCTTCATACTTCTACTACAATGC | PameOR47-R | CATCGCTTACAGACTCGTA |
| PameOR48-F | TCAGATTTCACTCGGCTAA | PameOR48-R | CATACGAACCGCAGATTG |
| PameOR49-F | TGAATGCTGGACAGATGT | PameOR49-R | TGTGCTTGTTGTTACAGATAG |
| PameOR50-F | ATTCGTCTACTGCTGGTT | PameOR50-R | AATGACAGAGGAGTTCCAA |
| PameOR51-F | TGAATGGATTGGGACTTCT | PameOR51-R | TAGAATAAGTGACTGGAAGGAT |
| PameOR52-F | GCTTCGGAAGTATGTTATCTC | PameOR52-R | CATATTCATAAGTGTAGCATTCG |
| PameOR53-F | GTGTGGAATGCTATTGGTT | PameOR53-R | CGCCTTGAAGGATGTCTA |
| PameOR54-F | TGCCTCTCATTCGTGTTA | PameOR54-R | TCTTGTATCATGCTCATCATC |
| PameOR55-F | AAGGAGTATGACGCTGTT | PameOR55-R | AAGGTAGAGGTGTTGAGAG |
| PameOR56-F | CATCCGTCATCAGTCCATA | PameOR56-R | ACAGAGTCACTAGAAGTATCAG |

| | | | |
|---|---|---|---|
| PameOR57-F | TCTGTTAGTCGTCTTTCTGT | PameOR57-R | CAAGCCCAAGCAATATCTC |
| PameOR58-F | CATGATGATAATGGCGAGG | PameOR58-R | TGACTGAACTAGCACTCC |
| PameOR59-F | GCCATCTTGTGTAGACTCT | PameOR59-R | CGCTGAACTTACATATTACATAAC |
| PameOR60-F | CACAAATGAACAACTAAGACAAA | PameOR60-R | GCCATCAACTTCGTAACTT |
| PameOR61-F | GTCTAACAGCACACTCATAAG | PameOR61-R | ATTTCACTTTCAATCACTGGAT |
| PameOR62-F | GCTTATGCTTCCGATTGG | PameOR62-R | CTCTTCTCGCTCTTGTCT |
| PameOR63-F | ACGAGGAGTAACAGGATTC | PameOR63-R | GGTGACCAAGACTTGATTG |
| PameOR64-F | CATTGTTCCAGTCTCTAATTCT | PameOR64-R | TCAGTGTAAATCTTCATTCTTGT |
| PameOR65-F | GAATCAAACAGAACAAAGGAAAT | PameOR65-R | CATAATACTGGTTGCGTCTT |
| PameOR66-F | CGCAGTATCATCTTCATCATT | PameOR66-R | TTCCTCGTTCTTATCCTTCA |
| PameOR67-F | ACTTCATCTGTGTCAACCT | PameOR67-R | CGAAGGAAGCAGAGTGATA |
| PameOR68-F | TTCCTATTACGAATGAAACTCTG | PameOR68-R | GCATACTTAATCAGTGTCACATA |
| PameOR69-F | CGCCAGGTCTAATAAGGAA | PameOR69-R | TTTCTGTAGCCGTTAATGTTT |
| PameOR70-F | ATACGCCAACATCAGGAG | PameOR70-R | CAGAAAGAAGCCAAGGAAC |
| PameOR71-F | CTTGGGCTTGTCATCATG | PameOR71-R | CTTCTTCATACAGTTGCTTCA |
| PameOR72-F | ATGTGCGTCGTTATGTTC | PameOR72-R | GCTCTCCTGTATAATATCTGTTC |
| PameOR73-F | GTATTATGAACCAGTCTCTATCG | PameOR73-R | TATTCGTTGTCCGTGTCT |
| PameOR74-F | TGCTGCTGCTACTATGAG | PameOR74-R | ATTGCCTACAGATTCGTACT |
| PameOR75-F | AGAAGCACTTGGTACTCAT | PameOR75-R | GCCCATTTGACAGTTGATAT |
| PameOR76-F | AGGCACCAGAAGACAATT | PameOR76-R | AAGCATTGGAGCATATCATAA |
| PameOR77-F | CATTCGTATATTGCGGCTT | PameOR77-R | GAGTTCCAATCCAATCAGAG |
| PameOR78-F | AGTTCCTTCCGAATGTAGTT | PameOR78-R | CGTAACCGACTTCTGACT |
| PameOR79-F | TCTTACCAACGATGTATCCT | PameOR79-R | GAGACGGGAACGAATTTG |
| PameOR80-F | TCACATAGTACAGCACCAT | PameOR80-R | CAGCCATCATTAAACTCATAGA |
| PameOR81-F | AACCTGGATTCGTTATGGA | PameOR81-R | TAACCTGTCGTCTTCACA |
| PameOR82-F | TTCATGTACTGTATCTGTATTGC | PameOR82-R | GAAATCATTGAGGTAATCTTGC |
| PameOR83-F | AACAAACCTCACTACTCAGA | PameOR83-R | CGTAATATCACCAACCTCAAG |
| PameOR84-F | ACGACAGGAATCAGAAGAT | PameOR84-R | GCCGTATCAGGTACAGAT |
| PameOR85-F | GGAACGCCTGTTACATATC | PameOR85-R | TGGAGATTGGAACGACTT |
| PameOR86-F | ACATATTATACACGGTAATCATCG | PameOR86-R | AGAGAGATCGCTGAAACG |

| | | | |
|---|---|---|---|
| PameOR87-F | CGCTTCATCATTTCAATAACAA | PameOR87-R | GAACAACGACATAGACTCATT |
| PameOR88-F | AGCATCCTTGGTGTGTTA | PameOR88-R | AATCTCATTGACTGGTCGTA |
| PameOR89-F | AAGATAGCCTGCTGAGTATT | PameOR89-R | GATGGTGTCGTATACATTCATT |
| PameOR90-F | TGAATCAGGTCGTTGGAG | PameOR90-R | CAGGCGTTTCTTTCTTGT |
| PameOR91-F | ACAGTCAACGAGGTAATCTAT | PameOR91-R | AATAGTGAAGTAGGCGTACA |
| PameOR92-F | CAGTACAGTGCGGTCTTA | PameOR92-R | TGTGGAGTGATTTCTGATGA |
| PameOR93-F | AGAAATTCGGCAGGTGTA | PameOR93-R | TATTCATAGTATCCTCCAGCAA |
| PameOR94-F | GGAATCTTCAACGACTTATCTT | PameOR94-R | AGTCACTGTTGGGAACTAA |
| PameOR95-F | GGATGTCATACGAATGTCTG | PameOR95-R | AGAACTGGAAGAAGAGAATAATG |
| PameGR1-F | TGGACAGTTGGCATCTTA | PameGR1-R | ATCGTGTTGAAGGTGACT |
| PameGR2-F | CCGTAACTTAGTCTCGTGTA | PameGR2-R | ACAACTCCTTCTGCTTGA |
| PameGR3-F | TCTATCATGCCTTACACAGT | PameGR3-R | TAGGACATCCAATTCTTACGA |
| PameGR4-F | TCAACCTTGGTGGATATGT | PameGR4-R | GGCTCCTATTCTGAACTGA |
| PameGR5-F | ACCACCTACCTCATCATTC | PameGR5-R | TTGCCTCAGTCAGATTGA |
| PameGR6-F | ATAGTTGCCGTCTCTCTTT | PameGR6-R | CTGTTGACACCTGATGGA |
| PameGR7-F | TGAGATGGAACAACTACAGA | PameGR7-R | GTAACAGAGAATATACGCAGAC |
| PameGR8-F | ACACTCAAGACAGATTGTTATG | PameGR8-R | TCATCACCATCATCGTCAT |
| PameGR9-F | TGAGCATTCCAGAGTGTT | PameGR9-R | AGCAAGGCAATCATTATATCC |
| PameGR10-F | TCAACTTCGGTGGATACG | PameGR10-R | ATGATGGTGTTACAGTAGTGA |
| PameGR11-F | GTTGCTGGATGTGTTATCTG | PameGR11-R | GCTCTCGGTCTCTGAATT |
| PameGR12-F | AACAAGCAACACGACAAG | PameGR12-R | ACACTCATTCATGTAGACCTAT |
| PameGR13-F | GTTCTCCAATGATAAGCAAGTA | PameGR13-R | GTAGGACAACCAGGTAGG |
| PameGR14-F | CTGTGGAAGGATAGAAGGAA | PameGR14-R | GTGCTCGTAAGAATGATGAA |
| PameGR15-F | CTGCTGAGTCCTGATCTT | PameGR15-R | TATAGAATGTAGTGTTGTCATGTC |
| PameGR16-F | TGGATTTGTGCTGTTTCG | PameGR16-R | TTCTCTTGGATGCTGACTT |
| PameGR17-F | GGCTGAAGATACCTGGAA | PameGR17-R | CGTAATGCTGTTAAGGAGTT |
| PameGR18-F | TGTTCTTCACCATCCAGAT | PameGR18-R | CATTTCCATTTCTTCAAGTTTCT |
| PameGR19-F | AAGTCTGATCTTGGTCCTT | PameGR19-R | TTAGTCGGCATGTCTTAATATAG |
| PameGR20-F | CAACACAACTCTAACTTATGGA | PameGR20-R | GCGGATGAATTAGCGAAA |
| PameGR21-F | TTACAAGATGTTATGGCTAAGG | PameGR21-R | AGATTATGGCACTGGAGAA |

| | | | |
|---|---|---|---|
| PameGR22-F | GCTGGTAATAATAGTGGTTCAT | PameGR22-R | CAGTCGCTTGTTCCATTG |
| PameGR23-F | TGGAATGGGAGAACTTAGAG | PameGR23-R | ATATCAGGAAGGCTGTCAG |
| PameGR24-F | GTCGTTTGGCAGTAATTTATAC | PameGR24-R | TTCTATAGTGCTCTCCATAGG |
| PameGR25-F | TGACAATGACTGCCACAT | PameGR25-R | CTGCTACCTCTGACAACA |
| PameGR26-F | TTGTCCCTTCGTGTCTTT | PameGR26-R | GCACCTCTAGTTCTTCTGA |
| PameGR27-F | GTGCTTACTGCCATGAATT | PameGR27-R | CGTATGTGATAATTGTGCCA |
| PameGR28-F | TCAGGATAGAGTTCATCACAT | PameGR28-R | GGAAGTCAGACCAGAGATT |
| PameGR29-F | GAGGCAACATTACAACTTATTATC | PameGR29-R | CACGAATGACAAGAAGTAGTAT |
| PameGR30-F | ACAGTACGCCAGTATAAGG | PameGR30-R | ATAAGAGCACAGAACAAGAAG |
| PameGR31-F | GTGCCATCTACATCTTATTCAA | PameGR31-R | CAACCGTAAGCAGAAGTG |
| PameGR32-F | TGGTCTTCTATGGAACTTCA | PameGR32-R | TCTTGTGCTTCAACTTGC |
| PameGR33-F | GGAAATCAACCTGGACTTG | PameGR33-R | GGGTACTGGGATCTCATC |
| PameIR1-F | TTGTTGTGAAGAATGTGTTAGA | PameIR1-R | ATGATGGCACGATACCTT |
| PameIR2-F | AGAGGAGAATGGATCTACAAG | PameIR2-R | AGTCAGCAGTGAAGTTAGT |
| PameIR8-F | CTGTTCGCAAGACTTCAC | PameIR8-R | TGTTCTGAGCACTGTAGG |
| PameIR3-F | TCTTGTTCTTCTGGCATCA | PameIR3-R | GAGTCTTGGCGTTCTTAAC |
| PameIR4-F | GCTGCTGTAGTGATAATAGTG | PameIR4-R | TACTTCCGCCTTGCTTTA |
| PameIR5-F | GGACAGTAACAGGCTCTC | PameIR5-R | GGTATGCGAACTCAAGTG |
| PameIR6-F | CCTCACTCACAACTTCCA | PameIR6-R | CTCAGATACTCCATAGACTCTT |
| PameIR7-F | TAATACAGGACATCGCAGAA | PameIR7-R | CCAGGATGAACAAGATAGGA |
| PameIR9-F | GGTGAATCTACTGGCAAAG | PameIR9-R | GCTTCTTCCACAACCTTC |
| PameIR10-F | GGAGAAGTTATGAATGGCAAT | PameIR10-R | TGGTGTAAGGAATCGTAAGA |
| PameIR11-F | ACAACCACAGTAACTTCAAG | PameIR11-R | GTAATTGCTAACGCTATACCA |
| PameIR12-F | TGTGCTACTCCTTTGCTAT | PameIR12-R | GTCTCCAGATTCTTCTCGTA |
| PameIR13-F | AGCATACAGGAGCAACAT | PameIR13-R | TCAATGAACAGAATCAGTACAG |
| PameIR14-F | CCTTGGTGCTCTTCGTAG | PameIR14-R | AAGTCGGTGAATGGAATGTTA |
| PameIR15-F | CATCGCTTTACGGATTACAG | PameIR15-R | ATGGAGCCTTGAGAAGTC |
| PameIR16-F | ATGTATTCCTGTCCGTTGAG | PameIR16-R | GTTCTGGAAGACGCTGTT |
| PameIR17-F | TGAACAGAATCGTGGCTATA | PameIR17-R | CTACTTCTCATCGCAGGAT |
| PameIR18-F | TTCCAAGGAGCACAATAGC | PameIR18-R | TTCACATGGTAATTCTTGAGACA |

| | | | |
|---|---|---|---|
| PameIR19-F | TCTGTAACACGAAGGTAATTG | PameIR19-R | CGATAGGATAAGAATGCTGAC |
| PameIR20-F | GAGAACCCTCCTATCACAATA | PameIR20-R | CTGTAACGCATTCCATACTG |
| PameIR21-F | GCAGATGAACGACTACAAG | PameIR21-R | TTGAGAATACTATGACCAACTG |
| PameIR22-F | GTCCACACTACCGAAGAG | PameIR22-R | GAAGTTCACCGTTGCTAC |
| PameIR23-F | ATAGGCTTGAAGGCACTT | PameIR23-R | AATGTTCATTTAGCGTTTGTTT |
| PameIR24-F | GTTACTGCGTTGCTATGG | PameIR24-R | AACAAGTTCCTCCAAGTCT |
| PameIR25-F | CCAATATCCAAGCACAGAAT | PameIR25-R | CGCAACTAATCAGAACACTT |
| PameIR26-F | TCAGCGTATGGAACAACA | PameIR26-R | CAGAGACAGCATCAGGAT |
| PameIR27-F | GAGTGACGCCTTTGTTATC | PameIR27-R | TTCCTCGTGGTCTTGATT |
| PameIR28-F | GTTATCGCCGTCGTAATG | PameIR28-R | CGAGAAGGATGGTCATATCA |
| PameIR29-F | TAGGTGGATACTTTGCTGAA | PameIR29-R | GAGGAGTTGTCTGTTGGA |
| PameIR30-F | TCAACGATGTCAGTATTCATAC | PameIR30-R | GTCAGCCATAGGTCAGAT |
| PameIR31-F | GGAGAAGGACATGGTGAA | PameIR31-R | GATGTTGGCGGAGTAAGA |
| PameIR32-F | GAGGATGAGTTGTTGGTCT | PameIR32-R | ATTAGTGCGGTGTGGATAA |
| PameIR33-F | CTTACAGGCTACGGACTAA | PameIR33-R | GTGATAAAGACGATACGACAA |
| PameIR34-F | TATTGCCTTGCTGTCAGTA | PameIR34-R | CATTCCACTTCAACACTTGTA |
| PameIR35-F | TTCACCACATTCCAAGGA | PameIR35-R | TTCTTCGGTAGAGACAACTT |
| PameIR36-F | GCTCCTCACCTACCTCTA | PameIR36-R | CATCAGCACCAATACAGTTAA |
| PameIR37-F | GTGCCACTTAGTATATGAACAA | PameIR37-R | GAAGCCAGACCTCGTAAT |
| PameIR38-F | ATCTTGTCCATCCGCTTC | PameIR38-R | TTGTTCCTTGCTCACTGA |
| PameIR39-F | GCCTCCATTCATTATAGGATC | PameIR39-R | ACTCCACAGTAGCATTCAA |
| PameIR40-F | AAAGGAGAGGTGGATGTC | PameIR40-R | TCCCTGGTTCTTTGATGT |
| PameIR41-F | TATTCTTGGTCTTGTCGTCTA | PameIR41-R | GATGAAGGTAGCGGAGTAA |
| PameIR42-F | GCTCTGGTTCATGTTCTG | PameIR42-R | CGACGAGGTCTTGGATAG |
| PameIR43-F | CGCATGGAAGTTGTTGAT | PameIR43-R | ACGGTGACATTATAGTGAGT |
| PameIR44-F | TAGCAGGTTATCAGATGGAATA | PameIR44-R | GCAAGTGTTCTGTTTAGCA |
| PameIR45-F | CTGCTGAACGCCTACTAC | PameIR45-R | AGCGGGTCAGAAATATCC |
| PameIR46-F | ATCCATAGGCTTGCTTCT | PameIR46-R | AAGATAATCCACCACTGTAGA |
| PameIR47-F | GTCTGCTACATCCTCCTG | PameIR47-R | CTTCTGGTCTTCCGTGAA |
| PameIR48-F | ACTCTGCCCATATTCTTCTC | PameIR48-R | ACTCTCATCCTGTTCATCAT |

| | | | |
|---|---|---|---|
| PameIR49-F | GAATGGTTCGTGGAATGG | PameIR49-R | CGTCCTGACTTCAAATAGAG |
| PameIR50-F | GCTATTGGAGGATTAACGATG | PameIR50-R | ACCTTATTGAGACATACATACTTG |
| PameIR51-F | GCATTGGCATCTTGTTCA | PameIR51-R | GGTTGTTCCACTCGTAGG |
| PameIR52-F | TTGAGCATCATCAGAGTGT | PameIR52-R | TGTTGAGTAAACAACTAGCATAT |
| PameIR53-F | CAGATCCGATTACTACAAACC | PameIR53-R | ATCCACTACGAACATCCG |
| PameiGluR1-F | TCCACTGTCAACTTCTTCA | PameiGluR1-R | CCTTGTTCATGTCCATGTATT |
| PameiGluR2-F | CTTCACGATCCACTGCTA | PameiGluR2-R | TCCTTCTCTGTCTCTCTTTG |
| PameiGluR3-F | CAAGACATTCGTGGTGAC | PameiGluR3-R | GTAGTTGAAGCCCAGGAT |
| PameiGluR4-F | AAAGAAGGGCGACGAATT | PameiGluR4-R | GTCTCAAGGATGGTGATGA |
| PameiGluR5-F | CTGAGCATTGTGAAGGATG | PameiGluR5-R | GATACCCAGCGACATGAA |
| PameiGluR6-F | CACACTGACTCACTTACTTG | PameiGluR6-R | CTTCATACTCGCCAGGTT |
| PameiGluR7-F | AAGAAGCGTTCCAAGATTG | PameiGluR7-R | ATAGCCACTGGTCATCATT |
| PameiGluR8-F | CTATTCAAGGTGCCAACTG | PameiGluR8-R | CTGGTTCACGAGATAGTCA |
| PameRPL17-F | AGCGAGCAAACTGAAATG | PameRPL17-R | AGCAGTCGTTGAAGGTAA |
| PameARF-F | TTGATGGTTGGTCTGGAT | PameARF-R | CCTGAGTATTCTGAAAGTAGTG |
| PameActin-F | GCGTGACATTAAGGAGAAG | PameActin-R | CAGGAAGGAAGGTTGGAA |

F: Forward, R: Reverse

**Figure S1**

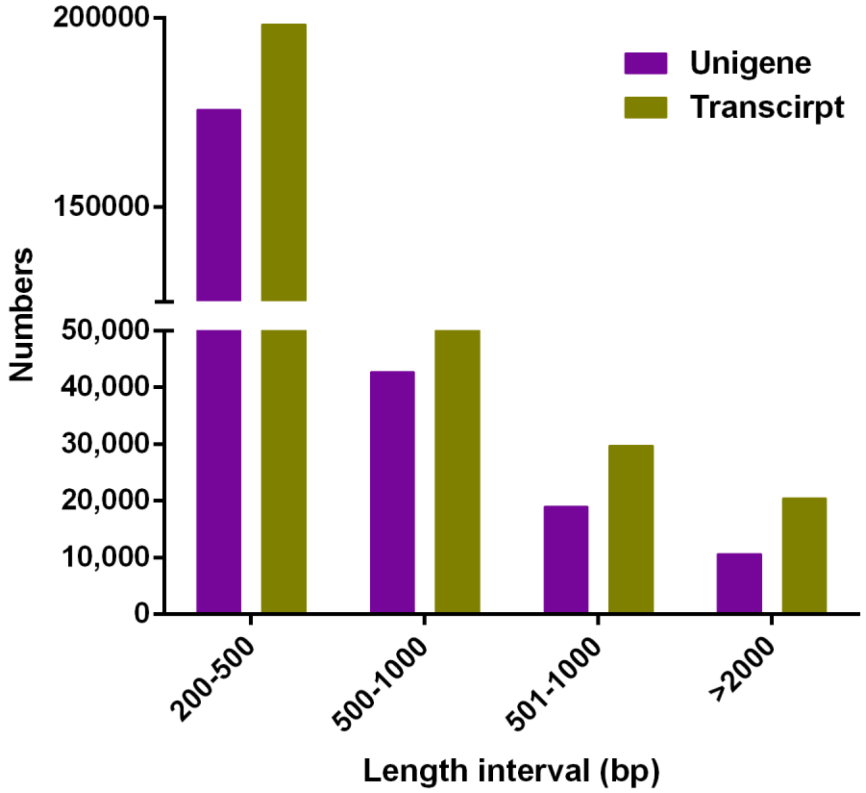

200000
150000
50,000
40,000
30,000
20,000
10,000
0
Numbers
Unigene
Transcirpt
200-500
500-1000
501-1000
>2000
Length interval (bp)
